# Reaching Out to Big Losers: How Different Types of Gamblers are Affected by a Brief Motivational Contact Initiated by the Gambling Provider

**DOI:** 10.1007/s10899-020-09978-7

**Published:** 2020-09-21

**Authors:** Jakob Jonsson, David C. Hodgins, Ingrid Munck, Per Carlbring

**Affiliations:** 1 Department of Psychology, Stockholm University, 106 91 Stockholm, Sweden; 2 Department of Psychology, University of Calgary, 2500 University Dr NW, Calgary, AB T2N 1N4 Canada; 3 Department of Education and Special Education, University of Gothenburg, P. O. Box 300, 405 30 Gothenburg, Sweden

**Keywords:** Behavioral feedback, Motivational intervention, Problem gambling, Prevention, Responsible gambling, Subtypes of gamblers

## Abstract

Telephone and letter-based motivational interventions with high expenditure gamblers have significant short and long term positive effects on gambling and use of responsible gambling tools. This report examines how different subtypes of gamblers, based upon patterns of play, are differentially affected. A randomized controlled trial design with three conditions (n = 1003 in each): feedback intervention by letter, telephone or a no-contact control condition. Subtypes of gamblers were derived by latent class analyses (LCA) based upon gambling behavior pre intervention. The participants were customers of Norsk Tipping gambling platforms. 1003 statistical triplets from the top 0.5% of customers based upon annual expenditure, matched on sex, age, and net losses. Primary outcome measure was gambling theoretical loss (TL), derived from the Norsk Tipping customer database. The LCA identified six subtypes: High Casino, High Sport, High Lottery, High Video lottery terminal (VLT), Lottery/Mix and Bingo/Casino. There were almost no differences in change in TL between the six subtypes of gamblers receiving the letter or telefone interventions respectively. However, the choice of contact by letter or telephone did have different effects for the different gambling subtypes. Sending a letter seems like a cost effective alternative to telephone contact for the High Lottery type, but telephone contact performs better for High Casino, High Sport and High VLT customers. Responsible gambling interventions can be improved by subtyping of gamblers.

**Electronic supplementary material:**

The online version of this article (10.1007/s10899-020-09978-7) contains supplementary material, which is available to authorized users.

## Introduction

Commercial gambling is a large industry with many multinational and national companies (Hodgins and Petry 2016). Although gambling is a recreational activity for most individuals, a significant proportion develop gambling-related problems and problematic gambling is considered a public health issue in many countries (Latvala et al. 2019; Wardle et al. 2019). Gambling disorder is characterized by loss of control and negative consequences (American Psychiatric Association 2013), and the prevalence for gambling disorder and its subclinical form is around 2–4% in most jurisdictions (Williams et al. 2012). High consumers are overrepresented among problem gamblers (Pallesen et al. 2020) and thus an obvious target for preventive efforts.

From prevalence studies and help seeking data, we find that certain gambling forms are more associated with gambling problems than others, particularly EGMs (electronic gambling machines such as slot machines and video lottery terminals; VLTs) and online casinos (Binde 2011; MacLaren 2016). This connection has been challenged with the argument that involvement in multiple gambling forms better explains gambling harm (e.g., LaPlante et al. 2013). Using longitudinal data, Binde et al. (2017) showed that problem gambling was more common among EGM, poker, casino and bingo players compared to other gambling forms, and that the relationship between gambling involvement and problem gambling is influenced by the forms of gambling played.

Many initiatives to reduce gambling-related harm by industry have been driven by government regulation. These initiatives are often labeled responsible gambling (RG). Research around RG has shown some promising results but is still in embryo (MacMahon et al. 2018, Tanner et al. 2017). Studies on measures such as pre-commitment and limit setting, self-exclusion, and messages/feedback on gambling behaviour have shown short term effects. In a recent systematic review including only studies with low or medium risk for bias found support for long term educational programs and personalized feedback had an impact on gambling behavior and that the follow-up period generally was short (Forsström et al. 2020). To our knowledge, only one study to date has focused on the longer term effect of providing feedback to high consumers (Jonsson et al. 2020).

Much of the research on RG has targeted measures developed for specific gambling forms (e.g., installing certain RG features on EGMs). A recent study found three distinct segments among gambling customers in Macau, and members of the casino gambling group were more likely to report symptoms of gambling disorder than the lottery and sociable gambler group members, suggesting a need for different interventions for different subtypes of gamblers (Nong et al. 2020). Studies of the differential effects of specific RG measures on different subtypes of gamblers are rare. In a study that provided the most intense playing customers at an online gambling site the opportunity to set voluntary spending and time limits, the largest effect for setting spending limits was seen for casino and lottery gamblers, and time limits affected the poker player most (Auer and Griffiths 2016). That study used a pre-post design and the groupings of gamblers were overlapping, in that many people engaged in more than one type of gambling. In another study, the same authors (Auer and Griffiths 2019) compared different statistical methods to predict limit-setting behavior from customers’ gambling, including participation and expenditure per gambling form. The expenditure per-gambling-variables were found to be predictive in one out of five statistical methods. This finding provides some support that RG measures could have different effects for different kinds of players. The current study addresses this research gap by examining how an RG intervention that targets high expenditure customers (Jonsson et al. 2019, 2020), differentially impacts gamblers that are participating in different forms of gambling. In the earlier analyses of the data, the high consumers were treated as a homogeneous group despite different patterns of involvement with different forms of gambling.

## Norsk Tipping Contacting Customer Intervention

**Fig. 1:**
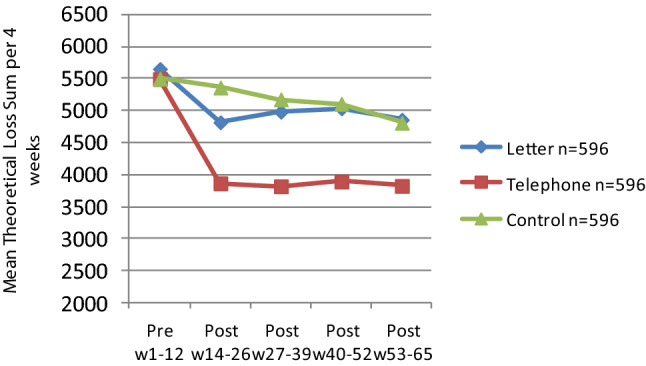
Mean profiles of theoretical loss sum per 4 weeks by contact type across pre-intervention week 1–12 to post-intervention periods week14–26, week 27–39, week 40–52 and week 53–65. *Note* Per protocol sample n = 596 × 3

Norsk Tipping (NT) is a state-owned gambling company with a broad gambling portfolio of online and land based forms of gambling.^1^ Since 2015, NT has been contacting high consumers by telephone and letter, aiming at encouraging the customers to reflect upon their gambling habits and guide them into taking action if desired and warranted. In a randomized controlled study with 1003 matched participants in each arm (telephone contact, letter contact and no contact control condition), the telephone intervention showed a clear effect short term (12 weeks) and the effect was stable over 12 months (Jonsson et al. 2019, 2020), see Fig. 1. Over 12 months, participants in the telephone group showed a 30% reduction in theoretic loss, the letter group 13%, and both outperformed the control group having a 7% reduction. Theoretical loss (TL) is the actual cost to the individual taking the house advantage for the type of gambling into account^2^ (Auer et al. 2012). Significantly more in telephone condition lowered their loss limits (one of the responsible gambling options for customers) during the year after intervention than the letter and control group.

## Objective

The study objective was to explore the heterogeneity in participation in gambling forms in the target population and how it might be related to the intervention results. In this analysis, we identify subtypes of gamblers through latent class analysis, based on variables measuring gambling intensity on different games during the 12 weeks before the study intervention. How these subtypes of gamblers are affected by the intervention is explored, which may have important implications for targeting these interventions. Analyses first empirically derived subtypes of gamblers based upon their baseline gambling involvements in different types of gambling. Overall post-intervention outcomes for these subtypes were then examined separately for participants receiving the letter and the telephone interventions. Finally, to explore how these interventions affect the different subtypes of gamblers the effects over one year were examined separately for each gambling subtype.

## Method

### Design and Participants

This is a secondary analysis of the Jonsson et al. study (2019, 2020) in which participants (n = 3009) were randomly selected from the top 0.5% of NT customers who had lost most money during the previous 12 months (N = 10,000). Statistical triplets (N = 3009 participants, 1003 triplets) were created using a statistical algorithm that matched on sex, age (+ / − 5 years) and net losses (+/− 10%). Each participant of the triplet was randomly assigned to one of the three contact type conditions; letter, telephone, or control.

In the original study, analyses were conducted on the entire sample (Intention to treat, ITT) and the sample of triplets who received the intended intervention (per protocol, PP). Triplets were included in the per protocol sample if individuals assigned to the telephone call and letter both received the assigned intervention. The per protocol sample was n = 1788, (596 triplets). In this study, the ITT sample was used to identify subtypes of gamblers and the per protocol sample, excluding the control group participants, was used in comparing effects of interventions for different subtypes of gamblers. Analyses of the telephone sample (n = 633) included all participants who were successfully contacted by telephone, whether or not their statistical twin assigned to the letter, received the letter. Similarly, analysis of the letter sample (n = 964) included all participants who received a letter (not returned to sender), whether or not their statistical twin assigned to the telephone sample received the telephone call.

The observation period covered 65 weeks. Pre-intervention period was week 1–12, followed by Intervention at week 13 and the 52 week post-intervention period (week 14–65). The interventions were completed between February and July 2017.

### Interventions

The participants in the telephone group were contacted by NT staff trained in motivational interviewing. Staff introduced themselves by saying,”NT has a campaign contacting their customers who have lost most money last year, and you are one of them. Do you have time to talk?”. The customers were asked to estimate their past year’s net result at NT and were asked if they wanted to hear the actual figure. The NT staff encouraged the customers to reflect on their gambling habits and they reinforced change talk using MI techniques. The NT staff informed participants about possible RG strategies, such as setting limits and taking breaks in play, and also helped the customer to initiate strategies if desired. The mean length of the calls were 6 min, ranging between 1 and 45 min.

The content of the letter was designed to mirror the telephone call as much as possible. It included an explanation as to why the customer had been sent the letter followed by personalized information on consumption, questions stimulating reflection, and information about possible RG actions if the customer wanted to make a change. The control group was not given any intervention.

### Ethical Statement

The study plan was approved by the Regional Ethical Review Board in Stockholm, Sweden.

## Measures and Data Collection

### Subtypes of Gamblers Variables

In order to assess the impact of heterogeneity of customers on outcome, we derived subtypes based upon individual patterns of gambling behavior during 12 weeks pre intervention. Gambling data for the ITT sample (n = 3009) were abstracted from the NT database, which was available for 100% of participants. The method used to identify subtypes of gamblers was Latent Class Analysis LCA, an application of mixture modeling (Masyn 2013), software used Mplus 8.2 (Muthén and Muthén 2018). LCA divides the total sample into a set of mutually exclusive discrete latent classes characterized by similar multidimensional patterns of gambling behavior (Bray 2007). Six indicators of gambling involvement were included in LCA measuring the mix of games played and intensity of play (Cunningham-Williams and Hong 2007). These indicators capture TL in each of the following games: Lottery, Sport, Casino, VLT, Scratch and Bingo. The three categories of intensity for each gambling form were (1) not played, (2) low and (3) medium–high, see Table 1. Details are provided in the supplement Table S.LCA1.

**Table 1:** Descriptive statistics for six different gambling forms played during pre-intervention week 1–12, n = 3009

Played gambling form			Pre-intervention week 1–12				
			TL sum per 4 weeks		Frequency distribution row% for categorized TL sum		
Play	n	Row %	Mean	Standard deviation	Not played code 1%	Low code 2%	Medium high code 3%
Lottery	2785	93	1548	1933	7	63	30
Sport	1946	65	1750	3191	35	50	14
VLT	1246	41	802	1581	59	20	21
Casino	1193	40	1251	2568	60	23	16
Scratch	707	23	119	577	77	20	3
Bingo	325	11	68	428	89	8	2

Currency is NoK

To compare alternative models, the LCA analyses ranged from 2–8 classes. The final 6-class solution was guided by weighing different criteria reported in Table 2 and interpretability of each solution. Following Nylund-Gibson and Choi (2018), the following criteria for class solution were used: (A) the highest entropy value 0.838 observed for 6 classes; (B) Minimum SSA-BIC is observed for 5 classes; and (C) Guided by the LRT test, which provides a *p*-value which indicates whether the k − 1 class model is rejected in favor of the k class model, the 4 classes solution gets support. Nylund-Gibson and Choi (2018) recommend the use of the most interpretable and sensible grouping within the range 4–6 classes. This resulted in choosing the 6-class solution. A detailed description of the LCA for subtype of gamblers is found in the supplement.

**Table 2:** LCA model fit of two up to eight class solution of subtypes of gamblers

# of Classes	BIC	SSA-BIC	AIC	Entropy	Lo-Mendell-Rubin adjusted LRT test	
					Value	*P*-value
1	28,873.97	28,835.84	28,801.86	–		
2	27,922.89	27,843.46	27,772.66	0.811	1043.358	0.000
3	27,538.64	27,417.90	27,310.29	0.791	468.859	0.000
4	27,242.69	27,080.64	26,936.21	0.774	376.566	0.000
5	27,212.43	27,009.07	26,827.83	0.77	119.471	0.684
6	27,257.58	27,012.92	26,794.86	0.838	52.42	0.940
7	27,310.73	27,024.76	26,769.89	0.795	45.317	0.764
8	27,371.63	27,044.36	26,752.67	0.745	38.423	0.773

### Primary Outcomes

Theoretic loss (TL) was the primary outcome variable. TL reflects the actual cost taking the house advantage into account (Auer et al. 2012). TL is calculated as wager x (1-pay back percentage) per game type. The measure of TL was defined as the consumption across 4-weeks periods expressed in currency NoK, in short ‘TL sum per 4 weeks’. The effect variable was changed in the mean TL sum per 4 weeks, from pre-intervention (weeks 1–12) to post-intervention periods (week 14–26, week 27–39, week 40–52, week 53–65).

### Secondary Outcomes

For the Telephone condition, telephone logs completed by the staff were used to measure call completion. Readiness to change, the participant’s motivation to change their gambling habits, was based on the NT staff ratings of the participant’s stage of change at the end of the telephone call.

### Statistical Analyses

Data was analyzed in SPSS, version 25 and Mplus version 8.2. A one way ANOVA was used to compare pre-intervention mean TL by subtype of gambler. To explore how the different interventions affected the subtypes of gamblers, the effects on change in TL over one year by gambling subtype were analyzed using separate one way between group ANOVAs for telephone and letter samples respectively, including post hoc Bonferroni-corrected *t*-test tests. To compare interventions per subtype of gamblers, mean TL sum per 4 weeks from pre- to post intervention across time are displayed as mean profiles in figures illustrating the effects for the gambling subtypes. These presentations of mean profiles are accompanied by separate repeated measure 2 × 5 ANOVAs with a Huyhn–Feldt correction to assess the interaction effect of intervention x time. Contrasts were used for post hoc tests, comparing each time period with the one preceding it. Chi square tests were used to analyze differences between subtypes of gamblers in response rate and change in motivation for the telephone intervention.

## Results

### Subtypes of Gamblers

As reported in Table 1, during the 12 weeks pre intervention 93% of participants played lottery, 65% sport betting, 41% VLT, 40% casino, 24% scratch and 11% bingo. The LCA identified six classes or subtypes, which are reported in Table 3 by their gambling intensity levels ranging from none up to high involvement in these activities (see details in note 2 to Table 3). The first class (n = 951) was labeled 'High Casino’ which was high in casino, and low in lottery, sport and VLT. The second class (n = 735) was ‘High Sport’ with high engagement in sport, while low in lottery and VLT. The third class (n = 641) was ‘High Lottery’, since lottery was high while involvement in both sport and VLT gambling was low. ‘High VLT’ was the fourth class (n = 403) with high engagement in VLT, but low in lottery and sport. ‘Lottery/Mix’ was the fifth class (n = 216) with players medium in lottery, and low in sport, casino and scratch. Finally, ‘Bingo/Casino’ was the sixth class (n = 63), which was medium in bingo, and medium/low in casino while low in lottery, sport and VLT. A post hoc power analysis revealed that Bingo/Casino was not large enough to detect an effect and draw any conclusions from in the following analysis, but the results are presented for descriptive reasons.

**Table 3:** Subtype of gambler profiles across different gambling forms based on within subtype mean theoretical loss sum per 4 weeks pre-intervention week 1–12

Gambling form	Subtype of gambler											
	High Casino n = 951		High Sport n = 735		High Lottery n = 641		High VLT n = 403		Lottery/Mix n = 216		Bingo/Casino n = 63	
	Mean TL	Intensity	Mean TL	Intensity	Mean TL	Intensity	Mean TL	Intensity	Mean TL	Intensity	Mean TL	Intensity
Lottery	768	L–L	669	L-L	3930	H	510	L–L	3112	M	630	L–L
Sport	1122	L–L	5046	H–H	338	L–L	380	L–L	460	L–L	321	L–L
VTL	587	L–L	283	L–L	282	L–L	3570	H	73	None	186	L–L
Casino	3587	H	80	None	73	None	73	None	559	L–L	1552	L
Scratch	119	None	18	None	1	None	5	None	1041	L–L	73	None
Bingo	34	None	3	None	0	None	9	None	50	None	2472	M
Total	6217		6099		4624		4547		5296		5234	

TL = TL sum per 4 weeks pre-intervention; Intensity = measure of mean TL levels of gambling. Currency is NoK

Notation for gambling intensity levels are based on within latent class mean TL ranging from 0 up to 5046 NoK which is divided into six intervals each about 1000 NoK wide. Mean TL levels are; None = '0 < = 167′, L–L = '167 to < = 1167′, L = '1167 to < = 2167′, M = '2167 to < = 3167′, H = '3167 to < = 4167′, H–H = ' 4167 to < = 5167'

**Fig. 2:**
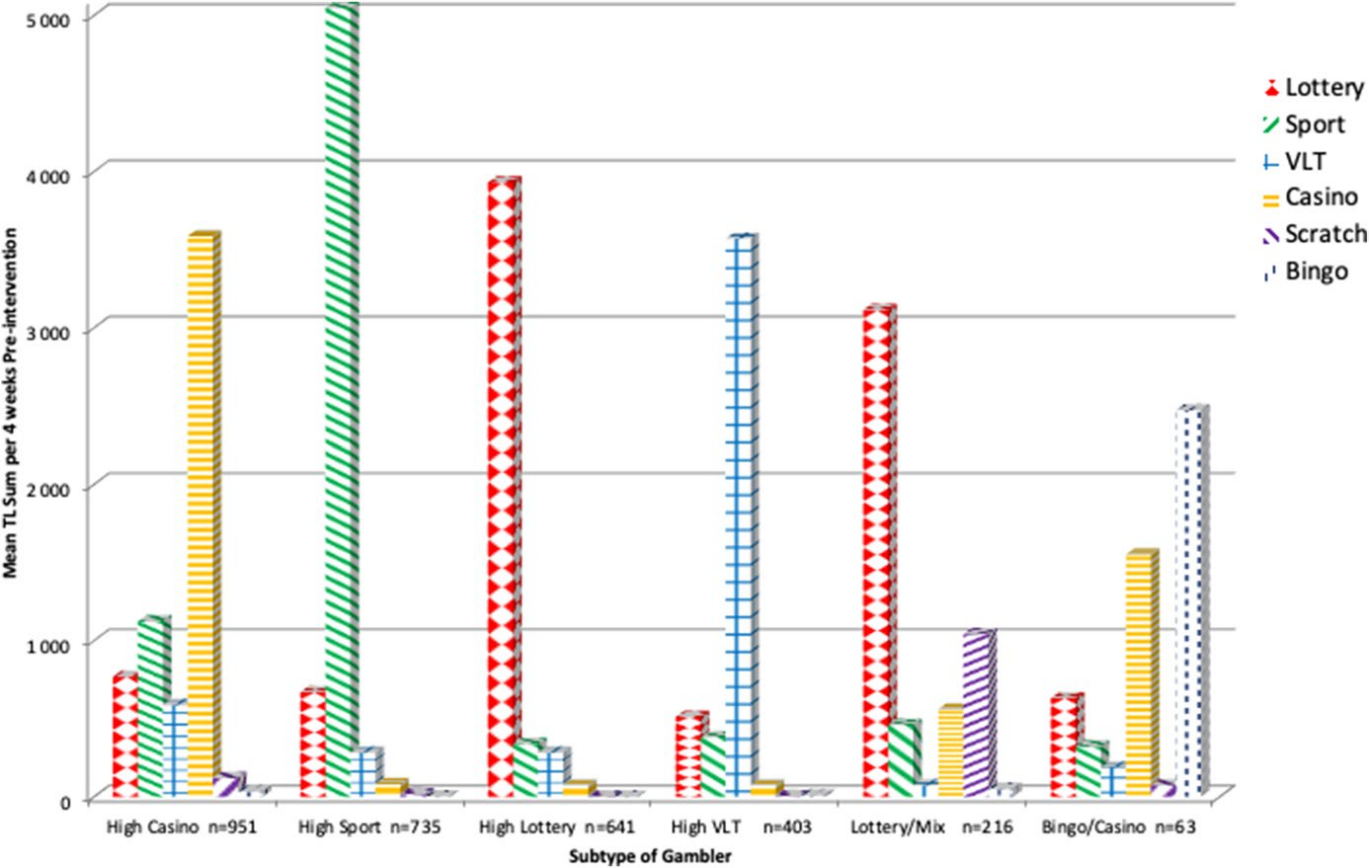
Type of gambler profiles across gambling forms*.* – mean for theoretical loss sum per 4 weeks pre-intervention across gambling forms within each gambling subtype. *Note* VLT = Video lottery terminal. n = 3009. Currency is NoK. See data and subtype description in Table 3

Table 3 and Fig. 2 displays the mean pre-intervention TL per gambling form for each of the six subtypes. A one way ANOVA showed that the pre-intervention mean total TL differed by subtype [F(5, 3003) = 25.43, *p* < 0.0001]. Post-hoc *t*-test comparisons using Bonferroni adjustment showed that the ‘High Casino’ group was significantly higher in TL than ‘High Lottery’ (*p* < 0.0001), ‘High VLT’ (*p* < 0.0001) and ‘Lottery/Mix’ (*p* < 0.01). ‘High Sport’ was significantly higher than ‘High Lottery’ (*p* < 0.0001) and ‘High VLT’ (*p* < 0.0001).

### Effect on Subtype of Gamblers by Telephone Intervention

A one way ANOVA for the telephone intervention group with completed calls showed that an overall difference for subtype of gamblers in change in TL post intervention [F(5, 627) = 2.51, *p* = 0.029]. Post hoc comparisons using Bonferroni adjustment showed only a trend toward significant differences between the subtypes, in which ‘High Casino’ decreased their theoretical loss more than ‘High Lottery’ (*p* = 0.064). There were no differences regarding response rate (participating in the telephone calls) between the subtypes [χ^2^(5, 1003) = 8.92, *p* = 0.112]. Regarding readiness to change, the proportion of each gambling subtype that was motivated (in the action phase) at the end of the call varied significantly by subtype [χ^2^(5, 633) = 38.93, *p* < 0.0001]. Least likely to be motivated were ‘High Lottery’ (26%) and ‘High Sport’ (38%) participants. Most frequently motivated were the ‘Bingo/Casino’ (62%), followed by ‘High Casino’ (58%), ‘High VLT’ (52%) and ‘Lottery/Mix’ (46%) participants.

### Effect on Subtype of Gamblers by Letter Intervention

A one way ANOVA showed no overall difference for subtype of gambler in change in TL post intervention [F(5, 958) = 1.78, *p* = 0.115]

### Effect of Letter and Telephone Intervention on Different Subtypes of Gamblers

**Fig. 3:**
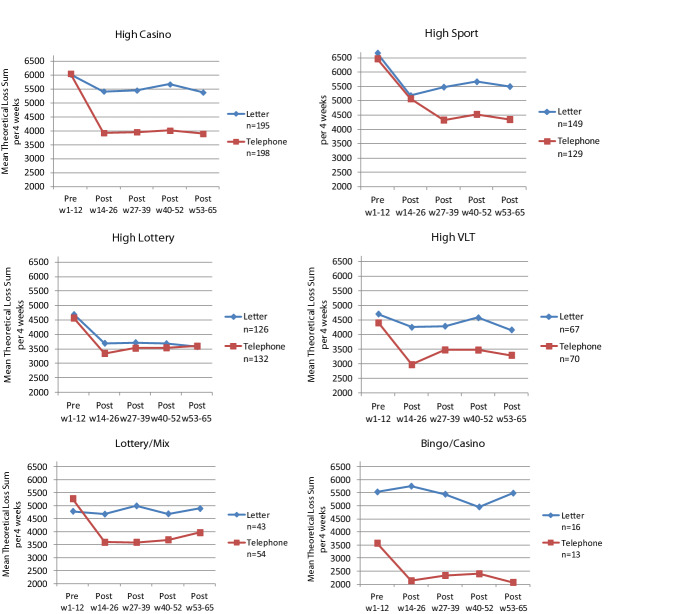
Mean theoretical loss sum per 4 weeks for subtypes of gamblers by letter and telephone intervention across pre-intervention week 1–12 and post-intervention periods for week 14–26 up to week 53–65. *Note* Per protocol samples; High Casino n = 393, High Sport n = 278, High Lottery n = 258, High VLT n = 137, Lottery/Mix n = 97 and Bingo/Casino n = 29. Currency is NoK

As seen in Fig. 3, different subtypes of gamblers responded differently to letter and telephone interventions. Statistical comparisons are provided in Table 4. For ‘High Casino’, there is a time and interaction x time effect, post hoc tests using contrasts show that the interaction effect is significant between pre-intervention and the first post period where telephone performs better. For ‘High Sport’, there is a time effect but no overall intervention x time effect, post hoc tests reveal a time effect between the pre and the first post period and a time x intervention effect between post the first and the second time periods where telephone performs better. For ‘High Lottery’, there is a time effect but no intervention x time effect, thus showing no difference between the letter and telephone intervention. For ‘High VLT’, there is a time effect but no overall intervention x time effect, post hoc tests reveal a time effect between the pre and the first post period and an intervention x time effect between pre-intervention and the first post period where telephone performs better. For ‘Lottery/Mix’, there is a time and interaction x time effect, post hoc tests using contrasts show that the interaction effect is significant between pre-intervention and the first post period where participants in the telephone condition have better outcomes.

**Table 4:** Two-way ANOVA with repeated measurement for time of theoretical loss sum by 4 weeks across five time points and by contact types letter and phone

Source	General linear model test statistics by subtype of gamblers																	
	Huynh–Feldt test of within-subjects effects																	
	High Casino			High Sport			High Lottery			High VLT			Lottery/Mix			Bingo/Casino		
	df	F	Sig.	df	F	Sig.	df	F	Sig.	df	F	Sig.	df	F	Sig.	df	F	Sig.
Time	3.7	14.90	0.000	3.0	11.20	0.000	3.6	28.73	0.000	3.4	4.48	0.003	3.3	4.10	0.005	2.9	0.69	0.560
Time*contact Type	3.7	5.16	0.001	3.0	1.68	0.171	3.6	0.57	0.668	3.4	1.14	0.334	3.3	4.22	0.005	2.9	0.67	0.571
Error (time)	1450.6			818.1			933.2			463.8			315.1			79.6		
Test of within-subjects contrasts																		
Time by level																		
w1-12 vs w14-26	1	50.04	0.000	1	22.71	0.000	1	67.69	0.000	1	17.28	0.000	1	9.61	0.003	1	1.22	0.280
w14-26 vs w27-39	1	0.03	0.865	1	0.87	0.352	1	0.72	0.398	1	1.99	0.161	1	0.58	0.449	1	0.01	0.935
w27-39 vs w40-w52	1	0.40	0.529	1	0.66	0.416	1	0.01	0.921	1	0.35	0.558	1	0.25	0.615	1	0.40	0.534
w40-52 vs w53-65	1	1.03	0.310	1	0.66	0.416	1	0.07	0.789	1	2.13	0.147	1	1.32	0.254	1	0.03	0.871
Time*contact type by level																		
w1-12 vs w14-26	1	15.52	0.000	1	0.02	0.882	1	0.71	0.401	1	4.65	0.033	1	7.40	0.008	1	2.27	0.144
w14-26 vs w27-39	1	0.00	0.990	1	4.76	0.030	1	0.42	0.518	1	1.49	0.224	1	0.59	0.443	1	0.15	0.703
w27-39 vs w40-w52	1	0.14	0.704	1	0.00	0.981	1	0.05	0.826	1	0.37	0.545	1	0.96	0.329	1	0.68	0.416
w40-52 vs w53-65	1	0.20	0.656	1	0.00	0.974	1	0.72	0.398	1	0.34	0.563	1	0.03	0.864	1	0.49	0.490
Tests of between-subjects effects																		
Intercept	1	941.07	0.000	1	325.53	0.000	1	809.75	0.000	1	470.08	0.000	1	278.47	0.000	1	93.03	0.000
Contact Type	1	14.13	0.000	1	1.61	0.206	1	0.35	0.553	1	5.73	0.018	1	2.20	0.141	1	12.67	0.001
Error	391			276			256			135			95			27		

Separate Analyses for Subtypes of Gamblers (correspond to Fig. 3)

Per protocol samples; high casino n = 393, high sport n = 278, high lottery n = 258, high VLT n = 137, lottery/mix n = 97 and bingo/casino n = 29. Currency is NoK

## Discussion

The objective of this secondary analysis of a trial of brief motivational interventions with high expenditure gamblers was to explore the heterogeneity in the target population of big losers and how different types of gamblers respond differently to the interventions. In the earlier analyses of the data (Jonsson et al. 2019, 2020), these high consumers were treated as a homogeneous group. In this paper, a LCA identified six classes based on involvement in different gambling forms: ‘High Casino’, ‘High Sport’, ‘High Lottery’, ‘High VLT’, ‘Lottery/Mix’ and ‘Bingo/Casino’.

There were no significant differences in change in TL between the six subtypes of gamblers receiving the telephone and letter interventions respectively. Thus, the interventions by telephone and letter seem to have a similar stable long-term effect on gamblers with different player profiles. These results are in contrast with those of Auer and Griffiths (2016), which may reflect methodological differences such as a shorter follow-up time and a focus on gambling forms rather than groups of gamblers.

Even though there were almost no differences in how subtypes of gamblers reacted to the letter and telephone interventions respectively, the choice of how to contact (by letter or telephone) have different effects on different gambling subtypes. Letter seems like a possible alternative to telephone regarding the ‘High Lottery’ type, but telephone performs better for ‘High Casino’, ‘High Sport’, ‘High VLT’ and ‘Lottery/Mix’. For ‘High Sport’, there is a delayed effect between the interventions, the telephone condition continues its decrease while letter does not. These differences might have implications for implementation, suggesting telephone as the only intervention that is effective for certain gambling types in the long term. These findings are preliminary and need further investigation in future research.

It is not surprising that the telephone intervention generally performed better given that it is a more personal and individualized interaction compared with a letter. It is unclear why this was untrue for lottery subtype gamblers. One possibility is that the lottery, arguably, involves less interpersonal interaction than the other types of gambling. Perhaps, these players are less interested or responsive to a personal contact. Lottery players as a group also have less problem gambling severity (Nong et al. 2020), and perhaps have less need to reduce their expenditures. Further research that includes a measure of problem severity for high expenditure players would provide a more nuanced interpretation of these results.

## Strengths

This study has several strengths. It has a high ecological validity with real customers, and with 100% coverage of the participants’ gambling at NT. It is well powered and incorporates many clinical trial design strengths. The study was conducted independently from NT, which had no design input or approval regarding any of the content published.

## Limitations

One weakness is the lack of information about the participants’ gambling elsewhere. Although NT has almost a 80% market share on the regulated gambling market in Norway, participants could access additional gambling venues or sites. Any additional gambling might have affected the subtypes identified by the LCA. Future research could consider combining behavioral with self-report data to ensure that all gambling for each participant is captured both at baseline and follow-up. A final weakness is that we cannot know to what extent customers received and understood the letters they were sent.

## Future Research

This study needs to be independently replicated, and the applicability of these findings in other jurisdictions needs to be confirmed. Norway is unusual in that it is a highly regulated gambling market where the majority of an individual’s gambling can be behaviorally tracked. Registered play is a prerequisite for conducting this kind of research with a high quality of data collection. Registered play constitutes a solid base for much needed evaluation and research of RG. In other jurisdictions registered play may be in place for online gambling but rarely for most land based gambling.

Research on the efficacy of responsible gambling tools has shown promising results but that the quality of evidence is still poor due to limited rigorous evaluations (Forsström et al. 2020). We are far from having a good understanding of how different RG measures should be matched to different subtypes of gamblers. Contacting high consumers is one aspect of the duty of care for the customers and could be part of stepped care models needing more investigation and research. It could be the case that sending a letter (like in this study) to some groups of players would have no effect, and this could guide the choice of intervention level.

## Electronic supplementary material

Below is the link to the electronic supplementary material.

Supplementary file1 (DOCX 6259 kb)

## Footnotes

1 Slot machines in physical venues, on- and offline lotteries (except physical scratch tickets) and sports betting, football pools, online bingo, and online casino.

2 Example: If one bet 100 € on a game with 85% payback, the theoretical loss would be 15 € [100 x (1 − 0.85)].
