# Supplementary material for: Reaching Out to Big Losers: How Different Types of Gamblers are Affected by a Brief Motivational Contact Initiated by the Gambling Provider
Source: J Gambl Stud. 2020 Sep 21;37(2):387–401. doi: 10.1007/s10899-020-09978-7 (PMC8144152; doi:10.1007/s10899-020-09978-7)
Supplement: Supplementary file 1 — Supplementary file1 (DOCX 6259 kb) [file 10899_2020_9978_MOESM1_ESM.docx]

# Supplement: Reaching out to big losers - How different types of gamblers are affected by a brief motivational contact initiated by the gambling provider- Data preparation, statistical analysis and supplementary tables.

This report of a Norsk Tipping NT study is a supplement to the article titled "Reaching out to big losers: How different types of gamblers are affected by a brief motivational contact initiated by the gambling provider " (NT Paper III) by AUTHORS. It is a complement to two earlier published articles by the same authors. The purpose is to provide technical information about data preparation, statistical analyses and some supplementary tables referenced in NT Paper III.

**Content**

1. Introduction
2. The objective of searching for subtypes of gamblers
3. Type of gambler traced by Latent Class Analysis (LCA)

3.1 Data preparation and Mplus LCA input and output

3.2 Results from the six class LCA solution - Tables

3.3 Results from the six class LCA solution - ANOVA testing of gambling consumption across subtype of gambler

3.4 Results from the six class LCA solution - Diagrams for subtype of gambler profiles concerning consumption of different gambling forms

4. Testing differences in change in theoretical loss for subtype of gamblers between pre-intervention and post-intervention.

4.1 Effect on subtype of gamblers by telephone intervention

4.2 Effect on subtype of gamblers by letter intervention

5. Effect of letter and telephone intervention on different subtype of gamblers tested with

repeated measures ANOVA.

6. References

**List of figures reporting LCA results for type of gambler**

*Figure S.LCA1 (Figure 1 in article).* Mean profiles of theoretical loss sum per 4 weeks by contact type across pre-intervention week 1-12 to post-intervention periods week 14-26, week 27-39,

week 40-52 and week 53-65.

*Figure S.LCA2 (Figure 2 in article).* Subtype of gambler profiles across gambling forms*. m*ean for theoretical loss sum per 4 weeks pre-intervention across gambling forms within each gambling subtype.

*Figure S.LCA3 (Figure 3 in article).* Mean theoretical loss sum per 4 weeks for subtype of gamblers by letter and telephone intervention across pre-intervention week 1-12 and post-intervention periods for week 14-26 up to week 53 -65.

**List of tables reporting LCA results for type of gambler**

Table S.LCA1 Extended. *Descriptive Statistics for Six Different Gambling Forms During Pre-intervention Weeks 1-12, n=3,009. Currency is NoK.*

Table S.LCA 1 (Table 1 in article). *Descriptive Statistics for Six Different Gambling Forms Played During Pre-intervention Week 1-12, n= 3,009. Currency is NoK.*

Table S.LCA 2 (Table 2 in article). *LCA Model Fit of One up to Eight Class Solution of Subtype of Gamblers.*

Table S.LCA 3 (Table 3 in article). *Subtype of Gambler Profiles Across Different Gambling Forms Based on Within Subtype Mean Theoretical Loss Sum per 4 Weeks Pre-intervention Week 1-12.*

Table S.LCA 3 Extended. (Data for *Figure S.LCA2) Subtype of Gambler Profiles Across Different Gambling Forms Based on Within Subtype Mean Theoretical Loss Sum Pre-intervention Week 1-12 per 4 Weeks including Sum TL.*

Table S.LCA 3 alt. *Subtype of Gamblers Across Gambling Forms. Consumption in Total % for TL Sum per 4 Weeks Pre-intervention Week 1-12.*

Table S.LCA 4. *Classification of Mean Theoretical Loss Sum per 4 Weeks Per-intervention Weeks 1-12 Into Gambling Intensity Categories.*

Table S.LCA 5. *Frequency Distributions for the LCA Six Classes by Contact Type based on Pre-intervention Data.*

Table S.LCA 6. (Table 4 in article). *Two-Way ANOVA with Repeated Measurement for Time of Theoretical Loss Sum by 4 weeks Across Five Time Points and by Contact Types Letter and Telephone. Separate Analyses for Subtype of Gamblers. (correspond to Figure 3 in article).*

## 1. Introduction

This research study concern heavy gambling customers of the regulated gambling market in Norway who were provided with brief feedback either by telephone contact or though mailed information on their gambling losses across all the available types of gambling at Norsk Tipping NT. It has a broad gambling portfolio including physical slots, on- and offline lotteries and sports betting, online bingo, and online casino. They do not offer bonuses to their customers. All gambling at NT is identified (except physical scratch tickets) and registered to the individual gambler.

The NT study is a full randomized controlled trial with participants from the top half percent of NT's customers who had lost most money the last 12 months, the target population. Over a period of five months and in five iterations, 1,003 statistical triplets (n=3,009) were created using a statistical algorithm that matched on sex, age (+/- 5 years) and net losses (+/- 10%).

Each participant within a triplet was randomly assigned using a true randomization process within the SQL environment in the NT customer database to one of the three conditions; Telephone, Letter or Control. Each triplet had one participant assigned to each condition. The pre-intervention period covered 12 weeks, the interventions took place during week 13 (a follow-up booster contact took place during week 17) and the post-Intervention of 12 weeks covered week 14 - 25 reported in NT Paper I and the 12 months covered up to week 62-65 reported in NT Paper II. Both these articles have methodological supplements where more details about statistical analyses are available (Authors).

In order to compare the outcome for separate subtype groups developed in this article with the overall effects across one year analyzed in earlier articles we report the three contact type's mean TL sum pre and post in Figure S.LCA1 ( Figure 1 in article).

*Figure S.LCA1 (Figure 1 in article).* Mean profiles of theoretical loss sum per 4 weeks by contact type across pre-intervention week 1-12 to post-intervention periods week 14-26, week 27-39,

week 40-52 and week 53-65.

Note. Per protocol sample n=596 x 3.

## 2. The objective of searching for subtypes of gamblers

## The objective was to explore the heterogeneity in the target population and how it might be related to the results of the interventions. In the earlier analyses of the NT data (Authors), the high consumers were treated as a homogeneous group despite different patterns of involvement with different types of gambling. In this analysis, we will identify subtypes of gamblers through latent class analysis, based on variables measuring gambling intensity on different games during the 12 weeks before the study intervention. How these subtypes of gamblers are affected by the intervention will be explored, aiming at a better understanding about how to approach the customer about RG with the letter or telephone intervention.

## 3. Type of gambler traced by Latent Class Analysis (LCA)

In the first analyses of the NT data we assumed that big looser gamblers are a fairly homogenous group with similar intervention effects across different gambling behavior in terms of gambling form played. In an attempt to explore the multidimensionality in the target population for the NT study we will now proceed to analyze individual patterns of gambling behavior based on variables measuring gambling intensity on different games.

Latent Class Analysis LCA will be applied to divide the total sample into a set of mutually exclusive discrete latent classes characterized by similar multidimensional patterns of gambling behavior (Bray 2007). Variations in individual choices of game played will be classified based on a set of six indicators underlying the latent construct of type of gambler measuring the mix of games played and with what intensity (Cunningham-Williams & Hong 2007). These indicators capture theoretical loss in each of the following games, Lottery, Sport, Casino, VLT, Scratch and Bingo.

The LCA method used to identify these subtypes of gamblers, labeled 'type of gambler', is an application of mixture modeling (Masyn 2013). The analyses are performed in software Mplus 8.2 (Muthén & Muthén 2018) which is designed for statistical analysis with latent variables. The data analyzed is the full sample n=3,009 across the 12 weeks pre-intervention period week 1-12.

### 3.1 Data preparation and Mplus LCA input and output

**Step 1:** For each gambling form a new variable for gambling intensity was computed measuring the weekly theoretical loss TL pre-intervention across weeks 1-12, in short 'TL by gambling form pre per week'.

**Step 2:** Using visual binning in SPSS the frequency distributions for the six 'TL by gambling form pre per week' variables were divided each into 10 categories(C10) and then transformed into 3 category variables in order to capture the level of gambling for each gambling form. The 3 categories are defined by code 1 'no game', code 2 'Low' and code 3 'high' according to the recoding below. The new indicator 'I3' variables are labeled 'SportI3', 'VLTI3', ' BingoI3', 'ScratchI3', 'LotteryI3', 'CasinoI3'.

SPSS recode

RECODE Sport_C10 (1=1) (2=2) (3 thru Highest=3) INTO SportI3.

EXECUTE.

RECODE VLT_C10 (1=1) (2=2) (3 thru Highest=3) INTO VLTI3.

EXECUTE.

RECODE Bingo_C10 (1=1) (2=2) (3 thru Highest=3) INTO BingoI3.

EXECUTE.

RECODE Scratch_C10 (1=1) (2=2) (3 thru Highest=3) INTO ScratchI3.

EXECUTE.

RECODE Lottery_C10(1=1) (2=2) (3 thru Highest=3) INTO LotteryI3.

EXECUTE.

RECODE Casino_C10(1=1) (2=2) (3 thru Highest=3) INTO CasinoI3.

EXECUTE.0

In table S.LCA 1 descriptive statistics for the six different gambling forms, mean and standard deviation for TL sum week 1-12 pre-intervention, transformed into per 4 weeks, are reported for the number of individuals in the full sample n=3,009 that played each game. In addition, the frequency distributions for the TL indicator I3 variables defined above; Bingo is the less played game only 11% of the sample active to compare with the most popular game Lottery played by 93%. In terms of money spent it is Sport that is ranked highest with mean 1,750 NoK followed by Lottery mean 1,548 NoK while means per 4 weeks of money spent on Bingo and Scratch are quite low, 119 NoK and 68 NoK respectively as they are such infrequent games played in the total sample of 3,009 individuals.

Table S.LCA1 Extended. *Descriptive Statistics for Six Different Gambling Forms During Pre-intervention Weeks 1-12, n=3,009. Currency is NoK.*

Table S.LCA 1 (Table 1 in article) *Descriptive Statistics for Six Different Gambling Forms Played During Pre-intervention Week 1-12, n= 3,009. Currency is NoK.*

**Step 3:** Preparation of the dat-file for Mplus. The input file to Mplus consisted of ID followed by the six variables SportI3 VLTI3 BingoI3 ScratchI3 LotteryI3 CasinoI3

**Step 4:** Mplus runs corresponding to LCA analyses of 1 class up to 8 classes.

Reporting input output for six class solution

TITLE: NT_LCA_Type_of_Gambler number of classes eq x.

DATA: FILE IS NT_LCA_games_I3.dat;

VARIABLE:

NAMES ARE ID SportI3 VLTI3 BingoI3 ScratchI3 LotteryI3 CasinoI3;

USEVARIABLES ARE ID SportI3 VLTI3 BingoI3 ScratchI3 LotteryI3 CasinoI3;

CLASSES = c (x); /* c(x) ranging from 2 up to 8

NOMINAL = SportI3 VLTI3 BingoI3 ScratchI3 LotteryI3 CasinoI3;

IDVARIABLE IS ID;

ANALYSIS: TYPE = MIXTURE;

STARTS=500 20;

STITERATIONS=100;

OUTPUT: TECH1 TECH8;

Extract from Output

MODEL FIT INFORMATION Number of Free Parameters 77

Loglikelihood

H0 Value -13,320.431

H0 Scaling Correction Factor 1.0341

for MLR

Information Criteria

Akaike (AIC) 26,794.861

Bayesian (BIC) 27,257.582

Sample-Size Adjusted BIC 27,012.923

(n* = (n + 2) / 24)

Entropy 0.838

**Step 5:** Summary table of NT_Mplus_LCA runs with number of classes ranging from 1 to 8.

Table S.LCA 2 (Table 2 in article) *LCA Model Fit of Two up to Eight Class Solution of Subtype of Gamblers.*

*Note.* There are three different criteria for the choice of solution of number of classes for further analysis: 1. the highest entropy value 0.838 observed for 6 classes. 2. Min SSA-BIC is observed for 5 classes. 3. Guided by the LRT test, which provides a p-value which indicates whether the k-1 class model is rejected in favor of the k class model, the 4 classes solution is chosen. Nylund-Gibson, K., & Choi, A. Y. (2018).

**Step 6:** Summary of six classes solution

Table M1. Mplus output 6 classes: Counts and Percentages for Most Likely Latent Class Membership.

Table M2. Mplus output 6 classes: Classification Probabilities for the Most Likely Latent Class Membership

### 3.2 Results from the six class LCA solution - Tables

In the reporting below all results about the latent classes forming homogenous subtypes of gamblers are reported edited to suite the Paper III article about the 12 month follow-up tables and figures. The order of Gaming forms are ranked according to % game played from highest, Lottery 93% to lowest Bingo 11% played that game (see Table SLCA.1) The latent classes are ordered from highest level of consumption pre-intervention , sum of TL sum per 4 weeks, for High Casino to lowest for Bingo/Casino.

In order to further describe the gambling profile for each type of gambler across different forms of gambling the mean TL levels from table S.LCA 3 are classified as follows. The mean range for TL is 0 up to 5046 NoK which is divided into six intervals each about 1000 NoK wide. The six gambling intensity levels are; None, Low-Low, Low, Medium, High, High-High reported in Table LCA 3 (see notation in Note 2) and Table S.LCA 4.

In the extended table S.LCA 3 the total consumption for each type of gambler across the six gambling forms are reported.

Table S.LCA 3. (Table 3 in article and data for Figure 2 in article) *Subtype of Gambler Profiles Across Different Gambling Forms Based on Within Subtype Mean Theoretical Loss Sum per 4 Weeks Pre-intervention Week 1-12.*

*Note 1.* TL = TL Sum per 4 weeks Pre-intervention; Intensity = Measure of mean TL levels of gambling. Currency is NoK.

*Note 2.* Notation for gambling intensity levels are based on within latent class mean TL ranging from 0 up to 5,046 NoK which is divided into six intervals each about 1000 NoK wide. Mean TL levels are; None = '0 <=167', L-L= '167 to <= 1,167', L= '1,167 to <=2,167', M ='2,167 to <= 3,167', H ='3,167 to <=4,167', H-H =' 4,167 to <=5,167'.

Table S.LCA 3 Extended (data for *Figure S.LCA2 and Figure 2 in article).* *Subtype of Gambler Profiles Across Different Gambling Forms Based on Within Subtype Mean Theoretical Loss Sum Pre-intervention Week 1-12 per 4 Weeks including Sum TL.*

In order to get an overview of the total consumption of gambling across all gambling activities in the whole sample of n=3,009 during 4 weeks pre-intervention, it is allocated with 35% High Casino, 27% to High Sport, 18% to High Lottery, 11% to High VLT, 7% to Lottery/Mix and finally 2% to Bingo/Casino, see Table S.LCA 3 alt.

Table S.LCA 3 alt. *Subtype of Gamblers Across Gambling Forms. Consumption in Total % for Sum TL per 4 Weeks Pre-intervention Week 1-12.* Note. TL = TL Sum Pre-intervention per 4 weeks.

Table S.LCA 4. *Classification of Mean Theoretical Loss Sum per 4 Weeks Per-intervention Weeks 1-12 Into Gambling Intensity Categories.*

*Note:* Mean range is 0 up to 5,046, see Table S.LCA 3. Currency is NoK.

Table S.LCA 5. *Frequency Distributions for the LCA Six Classes by Contact Type based on Pre-intervention Data.*

| Subtype of Gambler | Contact Type | | | Total n |
| --- | --- | --- | --- | --- |
|  | Letter | Telephone | Control |  |
| High Casino | 314 | 345 | 292 | 951 |
| High Sport | 250 | 217 | 268 | 735 |
| High Lottery | 204 | 213 | 224 | 641 |
| High VLT | 131 | 133 | 139 | 403 |
| Lottery/Mix | 78 | 79 | 59 | 216 |
| Bingo/Casino | 26 | 16 | 21 | 63 |
| Total n | 1,003 | 1,003 | 1,003 | 3,009 |

The distributions of n's across contact type are as expected quite even as the contact type groups were randomized. Class 2 Bingo/Casino groups have too small n-values to be kept in the analyses other than for descriptive purpose. A post hoc power analysis in GLM SPSS procedure revealed that Bingo/Casino was not large enough to detect an effect and draw any conclusions from in the following analysis.

### 3.3 Results from the six class LCA solution - ANOVA testing of gambling consumption across subtype of gambler

In order to test within-class mean TL differences in levels of gambling consumption between the six subtypes of gamblers across the 12 week pre-intervention period one way ANOVA was used based on the ITT sample n=3,009. The total mean TL sum's across the gambling forms are reported in S.LCA3 for the six latent classes/subtypes. The result is F(5; 3,003)=25.43, p<.0001. Post-hoc t-test comparisons using Bonferroni adjustment showed that the High Casino group was significantly higher in TL than High Lottery (p<.0001), High VLT (p<.0001) and Lottery/mix (p<.01). High Sport was significantly higher than High Lottery (p<.0001) and High VLT (p<.0001).

### 3.4 Results from the six class LCA solution - Diagrams for subtype of gambler profiles concerning consumption of different gambling forms

To illustrate the mix of gambling forms played by different type of gamblers the within-class mean TL sums per 4 weeks pre-intervention are displayed for the six gambling form in Figure S.LCA2 with gambling form bars of within class means.


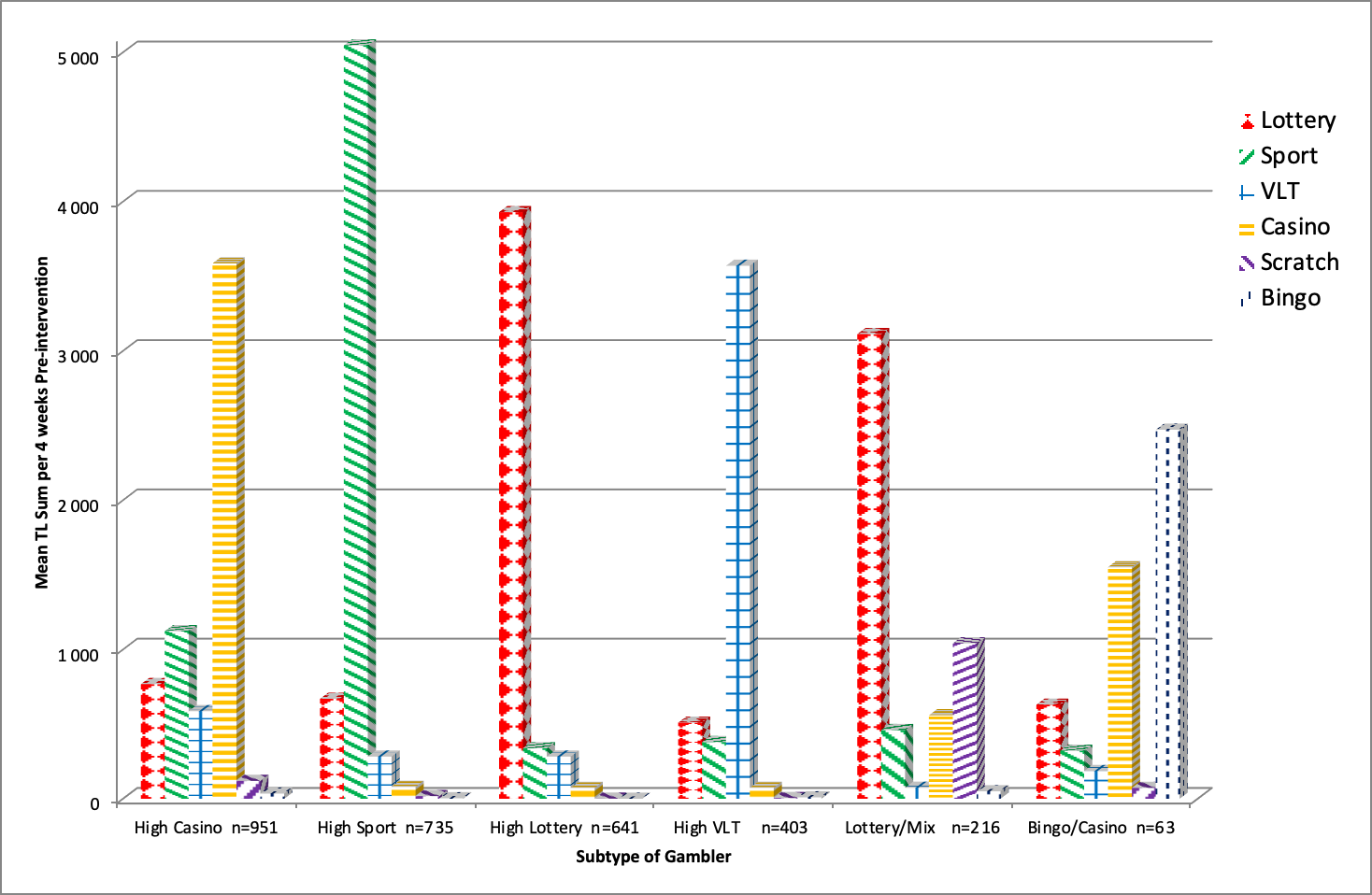


*Figure S.LCA2. (Figure 2 in article).* Subtype of gambler profiles across gambling forms, mean for theoretical loss sum per 4 weeks pre-intervention across gambling forms within each gambling subtype.

*Note.*  VLT=Video lottery terminal. n=3,009. Currency is NoK. See data and class description in Table S.LCA 3 (Table 3 in article).

## 4. Testing differences in change in theoretical loss for subtype of gamblers between pre-intervention and post-intervention

### 4.1 Effect on subtype of gamblers by telephone intervention

A one way ANOVA for the telephone intervention group with completed calls showed that an overall difference for subtype of gamblers regarding change in TL post intervention (F(5; 627)=2.51, p=.029). Post hoc comparisons using Bonferroni adjustment showed only a trend toward significant differences between the subtypes, in which High Casino decreased their theoretical loss more than High Lottery (p=.064). There were no differences regarding response rate (participating in the telephone calls) between the subtypes (F(5; 1,003)=8.92, p=.112).

### 4.2 Effect on subtype of gamblers by letter intervention

A one way ANOVA showed no overall difference for subtypes of gambler regarding change in TL post intervention (F(5; 958)=1.78, p=.115).

## 5. Effect of letter and telephone intervention on different subtype of gamblers tested with repeated measures ANOVA

To compare interventions per subtype of gamblers, mean TL from pre- to post intervention time periods are reported as mean profiles in Figure S.LCA3 illustrating the effects for subtypes. The presentation of mean profiles in diagrams are accompanied by repeated measures ANOVAs with a Huyhn-Feldt correction to reveal an interaction effect of time x intervention type. Contrasts are used for post hoc tests, comparing each time period with the one preceding it, Table S.LCA 6.

*Figure S.LCA3 (Figure 3 in article).* Mean theoretical loss sum per 4 weeks subtypes by letter and telephone intervention across pre-intervention week 1-12 and post-intervention periods for week 14-26 up to week 53 -65.

*Note1.* Per protocol samples; High Casino n=393, High Sport n=278, High Lottery n=258, High VLT n=137, Lottery/Mix n=97 and Bingo/Casino n=29. Currency is NoK.

In *Figure S.LCA3* and corresponding Table S.LCA 6 one outlier 'IDcustomer 1410' in the subgroup Bingo/Casino in the Letter invention at Time 3 post w27-39 was trimmed from TL value 38934 to 6142, the 5%trimmed mean in SPSS Explore.

Table S.LCA 6. (Table 4 in article). Two-Way ANOVA with Repeated Measurement for Time of Theoretical Loss Sum per 4 weeks Across Five Time Points and by Contact Types Letter and Telephone. Separate Analyses for Subtype of Gamblers; High Lottery, High Casino, High Sport, High VLT, Lottery/Mix and Bingo/Casino. (correspond to Figure 3 in article).

## 6. References

Bray, B.C. (2007). *Examining Gambling and Substance Use: Applications of Advanced Latent Class Modeling Techniques for Cross-sectional and Longitudinal Data.* Dissertation.

Cunningham-Williams, R.M. and Hong, S.I. (2007). A Latent Class Analysis (LCA) of Problem Gambling Among a Sample of Community-Recruited Gamblers. *The Journal of Nervous and Mental Disease,* 195 (11).DOI: 10.1097/NMD.0b013e31815947c1.

Field, A. (2009) *Discovering Statistics Using SPSS*. Third Edition. SAGE Publications: London.

Masyn, K. E. (2013). Latent Class Analysis and Finite Mixture Modeling. In P. Nathan and T. Little (Eds.), *The Oxford Handbook of Quantitative Methods* (pp. 551-611). New York, NY. Oxford University Press.

Muthén, L.K., Muthén, B.O. (2018). *Mplus User’s Guide.* Eight Edition. Los Angeles, CA: Muthén & Muthén.

Nylund-Gibson, K., Choi, A.Y.(2018). Ten Frequently Asked Questions About Latent Class Analysis. *Translational Issues in Psychological Science.*
